# Low utilization of confirmatory testing for tinea capitis by pediatricians at an academic center in New York, United States, 2005–2021

**DOI:** 10.3389/fped.2023.1297339

**Published:** 2023-11-17

**Authors:** Jonathan K. Hwang, Jeremy A. W. Gold, Amy S. Paller, Shari R. Lipner

**Affiliations:** ^1^ Department of Dermatology, Weill Cornell Medicine, New York, NY, United States; ^2^ Mycotic Diseases Branch, Division of Foodborne, Waterborne and Environmental Diseases, Centers for Disease Control and Prevention, Atlanta, GA, United States; ^3^ Departments of Dermatology and Pediatrics, Northwestern University Feinberg School of Medicine, Chicago, IL, United States

**Keywords:** tinea, tinea capitis, fungal infections, diagnosis, treatment

## Abstract

We retrospectively reviewed physician diagnostic and treatment practices for pediatric tinea capitis at an academic institution over 16 years, in assessing adherence with published guidelines. We demonstrate the need to increase utilization of confirmatory testing and systemic therapy, and call for directed pediatrician education towards these goals.

## Introduction

Tinea capitis (TC), a fungal scalp infection, is the most common childhood dermatophytosis worldwide (1). TC primarily affects children aged 3–14 years, particularly Black males, and is most often caused by *Microsporum* and *Trichophyton* species. In the United States, *T. tonsurans* is the most common causative species (2). American Academy of Dermatology guidelines (1996, most recent year) and the American Academy of Pediatrics Committee on Infectious Disease (2021 Red Book, most recent year) emphasize confirmatory testing of suspected TC and treatment with systemic antifungals (3, 4). Confirmatory testing is important given possibility of misdiagnosis, ineffectiveness of topicals against TC, and need for antifungal stewardship in an era of emerging antifungal-resistant dermatophytes (5). Because data on guideline adherence are lacking, we aimed to capture TC diagnostic and treatment practices.

## Methods

After Weill Cornell Medicine Institutional Review Board approval (22-09025241), Weill Cornell Medicine EPIC database was queried for patients 0–18 years diagnosed with TC (International Classification of Diseases-9 code 110.0, International Classification of Diseases-10 code B35.0) 10/1/2005-9/30/2021. Demographics, diagnosing physician specialty, diagnostic test(s) performed, and treatment(s) prescribed were described. Chi-squared tests compared diagnostic and treatment practices for dermatologists vs. pediatricians (*α* < 0.05).

## Results

**Figure 1:**
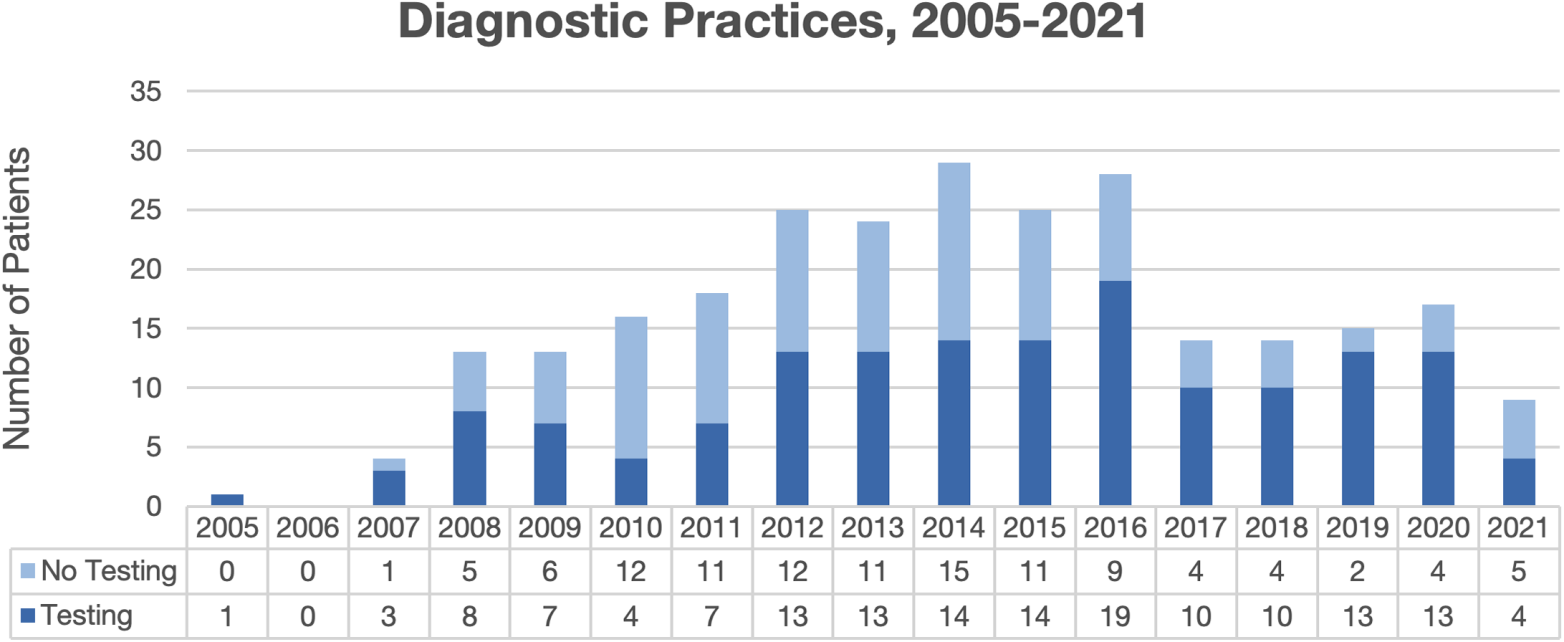
Diagnostic practices for patients with tinea capitis diagnoses from 2005 to 2021.

Overall, 265 total patient visits were included, comprising 239 patients, with average age 4.4 years, 56.5% male, and 70.8% Black (Table 1). Most were diagnosed by dermatologists (49.8%) or pediatricians (49.1%). Diagnostic testing was performed in 57.7% of encounters, most commonly fungal cultures (94.8%) and potassium hydroxide (KOH) preparation (54.9%) (Table 2). Testing was performed more often by dermatologists than pediatricians (96.2% vs. 20.0%, *p* < 0.00001), with testing practices by specialty relatively stable over the study period (Figure 1). Identified species included 77.1% *T. tonsurans*, 4.2% *T. rubrum*, and 2.1% *M. canis*. Systemic therapy was prescribed most often (92.6%), and more commonly by dermatologists than pediatricians (96.6% vs. 88.6%, *p* = 0.02). Common systemics were griseofulvin (86.7%) and terbinafine (16.0%), with terbinafine more often utilized by dermatologists (30.0%) than pediatricians (1.8%). Systemics besides griseofulvin were utilized more often 2014–2021 vs. 2005–2013 (24.4% vs. 11.2%, *p* = 0.01). Common topicals were ketoconazole (69.5%) and clotrimazole (20.7%), with clotrimazole only prescribed by pediatricians.

**Table 1:** Diagnosing physician specialty and demographics of patients with tinea capitis diagnoses from 2005 to 2021.

Encounters with tinea capitis diagnostic code (*n* = 265)		
Physician specialty		
Dermatology	132	49.8%
Pediatrics	130	49.1%
Emergency medicine	3	1.1%
Patient demographics (*n* = 239)		
Age at first encounter (average), years	4.4 (1 month−15 years old)	
<1	16	6.7%
1–4	119	49.8%
5–10	98	41.0%
11–17	6	2.5%
Gender		
Male	135	56.5%
Female	104	43.5%
Race		
Black	126	70.8%
White	46	25.8%
Asian	6	3.4%
Unknown	61	
Ethnicity		
Hispanic	50	25.9%
Not Hispanic	143	74.1%
Unknown	46	

**Table 2:** Diagnostic and treatment practices for patients with tinea capitis diagnoses from 2005 to 2021.

Diagnostic practices^a^	All (*n* = 265)^c^	Dermatology (*n* = 132)	Pediatrics (*n* = 130)	*P*-value^^^
Testing performed	153 (57.7%)	127 (96.2%)	26 (20.0%)	
Fungal culture	145 (54.7%)	120 (90.9%)	25 (19.2%)	
Potassium hydroxide preparation	84 (31.7%)	84 (63.6%)	0	
Scalp biopsy	1 (0.4%)	1 (0.8%)	0	
Wood's lamp	1 (0.4%)	0	1 (0.8%)	
No testing performed	112 (42.3%)^c^	5 (3.8%)	104 (80.0%)	
Deemed not diagnostic after testing	22 (8.3%)	15 (11.3%)	7 (5.4%)	
Performed confirmatory testing	153 (57.7%)	127 (96.2%)	26 (20.0%)	*p* < 0.00001
No confirmatory testing performed	112 (42.3%)^c^	5 (3.8%)	104 (80.0%)	
Treatment practices^b^	All (*n* = 243)^c^	Dermatology (*n* = 117)	Pediatrics (*n* = 123)	
Systemic antifungals	225 (92.6%)^c^	113 (96.6%)	109 (88.6%)	
Griseofulvin	195 (80.2%)^c^	85 (72.6%)	107 (87.0%)	
Terbinafine	36 (14.8%)	34 (29.1%)	2 (1.6%)	
Itraconazole	1 (0.4%)	1 (0.9%)	0	
Ketoconazole	1 (0.4%)	1 (0.9%)	0	
Fluconazole	1 (0.4%)	0	1 (0.8%)	
Topical antifungals	82 (33.7%)	47 (40.2%)	35 (28.5%)	
Ketoconazole	57 (23.5%)	39 (33.3%)	18 (14.6%)	
Clotrimazole	17 (7.0%)	0	17 (13.8%)	
Econazole	4 (1.6%)	3 (2.6%)	1 (0.8%)	
Terbinafine	2 (0.8%)	2 (1.7%)	0	
Miconazole	1 (0.4%)	0	1 (0.8%)	
Systemic corticosteroids	2 (0.8%)	1 (0.9%)	1 (0.8%)	
Topical corticosteroids	1 (0.4%)	0	1 (0.8%)	
Prescribed systemic and/or topical therapy	225 (92.6%)^c^	113 (96.6%)	109 (88.6%)	*p* = 0.02
Prescribed topical therapy only	18 (7.5%)	4 (3.4%)	14 (11.4%)	

^a^ No encounters utilized polymerase chain reaction testing or antifungal susceptibility testing.

^b^ Cases deemed not diagnostic after testing resulted are excluded from this section, as prescribed treatments were discontinued.

^c^ Three encounters in which patients were seen by emergency medicine physicians (all prescribed systemic griseofulvin only without confirmatory testing) were included in total count, but excluded from *p*-value calculations.

^^^ *P*-values were calculated using chi-square tests comparing dermatologists vs. pediatricians.

## Discussion

In this 16-year retrospective review of TC patients at Weill Cornell Medicine, virtually all dermatologists, but only one-fifth of pediatricians, performed confirmatory testing. In a national commercial database study of 3.9 million pediatric TC encounters, confirmatory testing was infrequent (21.9%), with dermatologists testing more often than pediatricians (51.0% vs. 16.4%, *p* < 0.01), suggesting similar testing practices between academic and community pediatricians (6). KOH preparations were documented in approximately two-thirds of dermatologist encounters and in no pediatric encounters. Low testing rates by pediatricians may be due to lack of recognition of importance of diagnostic confirmation emphasized by American Academy of Dermatology guidelines and American Academy of Pediatrics Red Book recommendations, long fungal culture turnaround times competing with prompt treatment initiation, and lack of training or required Clinical Laboratory Improvement Amendments certification for KOH examinations (3, 4, 6).

Consistent with TC treatment guidelines, most patients were prescribed systemic antifungals (92.6%). Using topical antifungals (creams, shampoos) alone is discouraged, due to lack of hair follicle root penetration. However, 11.4% of pediatricians prescribed topical therapy only, similar to the aforementioned commercial database study reporting a 10.1% rate for pediatricians, emphasizing the need for directed education (6). For systemic therapies, a systematic review of 38 TC clinical trials reported overall 92% and 72% complete cure rates for terbinafine and griseofulvin, respectively, terbinafine being more effective for *Trichophyton* and griseofulvin more effective for *Microsporum* species (7). Terbinafine is regarded as first-line treatment in the US, given vast predominance of *Trichophyton* TC and shorter treatment course (6–8 weeks) (7). However, since terbinafine was infrequently prescribed by pediatricians (1.6%), directed education is necessary.

Limitations include single-center design and small sample size. However, our study may accurately reflect US practices, given congruence with the commercial database study (5).

We highlight opportunities to increase utilization of confirmatory testing and systemic therapy, and call for directed pediatrician education towards these goals.

## Data Availability

The raw data supporting the conclusions of this article will be made available by the authors, without undue reservation.
